# Cancer nanomedicine: a review of recent success in drug delivery

**DOI:** 10.1186/s40169-017-0175-0

**Published:** 2017-12-11

**Authors:** Stephanie Tran, Peter-Joseph DeGiovanni, Brandon Piel, Prakash Rai

**Affiliations:** 1 Department of Biological Sciences, University of Massachusetts, Lowell, MA 01854 USA; 2 Department of Chemical Engineering, University of Massachusetts, 1 University ave, Lowell, MA 01854 USA

**Keywords:** Nanoparticles, Oncology, Clinical trials, Therapeutics, Combination treatment, Theranostics, MM-398

## Abstract

Cancer continues to be one of the most difficult global healthcare problems. Although there is a large library of drugs that can be used in cancer treatment, the problem is selectively killing all the cancer cells while reducing collateral toxicity to healthy cells. There are several biological barriers to effective drug delivery in cancer such as renal, hepatic, or immune clearance. Nanoparticles loaded with drugs can be designed to overcome these biological barriers to improve efficacy while reducing morbidity. Nanomedicine has ushered in a new era for drug delivery by improving the therapeutic indices of the active pharmaceutical ingredients engineered within nanoparticles. First generation nanomedicines have received widespread clinical approval over the past two decades, from Doxil^®^ (liposomal doxorubicin) in 1995 to Onivyde^®^ (liposomal irinotecan) in 2015. This review highlights the biological barriers to effective drug delivery in cancer, emphasizing the need for nanoparticles for improving therapeutic outcomes. A summary of different nanoparticles used for drug delivery applications in cancer are presented. The review summarizes recent successes in cancer nanomedicine in the clinic. The clinical trials of Onivyde leading to its approval in 2015 by the Food and Drug Adminstration are highlighted as a case study in the recent clinical success of nanomedicine against cancer. Next generation nanomedicines need to be better targeted to specifically destroy cancerous tissue, but face several obstacles in their clinical development, including identification of appropriate biomarkers to target, scale-up of synthesis, and reproducible characterization. These hurdles need to be overcome through multidisciplinary collaborations across academia, pharmaceutical industry, and regulatory agencies in order to achieve the goal of eradicating cancer. This review discusses the current use of clinically approved nanomedicines, the investigation of nanomedicines in clinical trials, and the challenges that may hinder development of the nanomedicines for cancer treatment.

## Background

Cancer is currently among one of the leading causes of deaths worldwide, with 1,688,780 new cases and 600,920 cancer deaths projected for 2017. Over the next 20 years, the number of new cases is projected to increase by about 70% [1]. Current treatments may include chemotherapy, radiation, and surgery, but the effects of these procedures may damage not only the tumor tissue but also normal tissue. Weinberg and Hanahan have described a set of six hallmarks of cancer, which may help distinguish characteristics between the normal and tumor tissue and perhaps provide better alternative treatments. These hallmarks include sustaining proliferative signaling, evading growth suppressors, activating invasion and metastasis, enabling replicative immortality, inducing angiogenesis, and resisting cell death [2]. Even in the absence of injury or development, cancer cells can maintain growth signals and continue proliferation. Normal regulators of cell growth and apoptosis are often inhibited. High levels of telomerase may help cancer cells maintain the integrity of their DNA and thus allow them to replicate infinitely. The formation of new blood vessels, or angiogenesis, is a method for cancer cells to obtain nutrients and remove waste. Cancer cells can also migrate to new sites and form new, secondary tumors. Two emerging hallmarks of cancer include reprogramming energy metabolism and evading immune destruction. Cancer cells have upregulated glucose transporter expression, and they tend to reprogram their metabolic pathway to “aerobic glycolysis”. This metabolic switch may allow the generation of nucleosides and amino acids, which facilitate additional growth and proliferation. Markers that T-lymphocytes use to recognize and destroy foreign or abnormal cells are not well-expressed by cancer cells, thus allowing them to avoid elimination by the immune system [2].

**Fig. 1:**
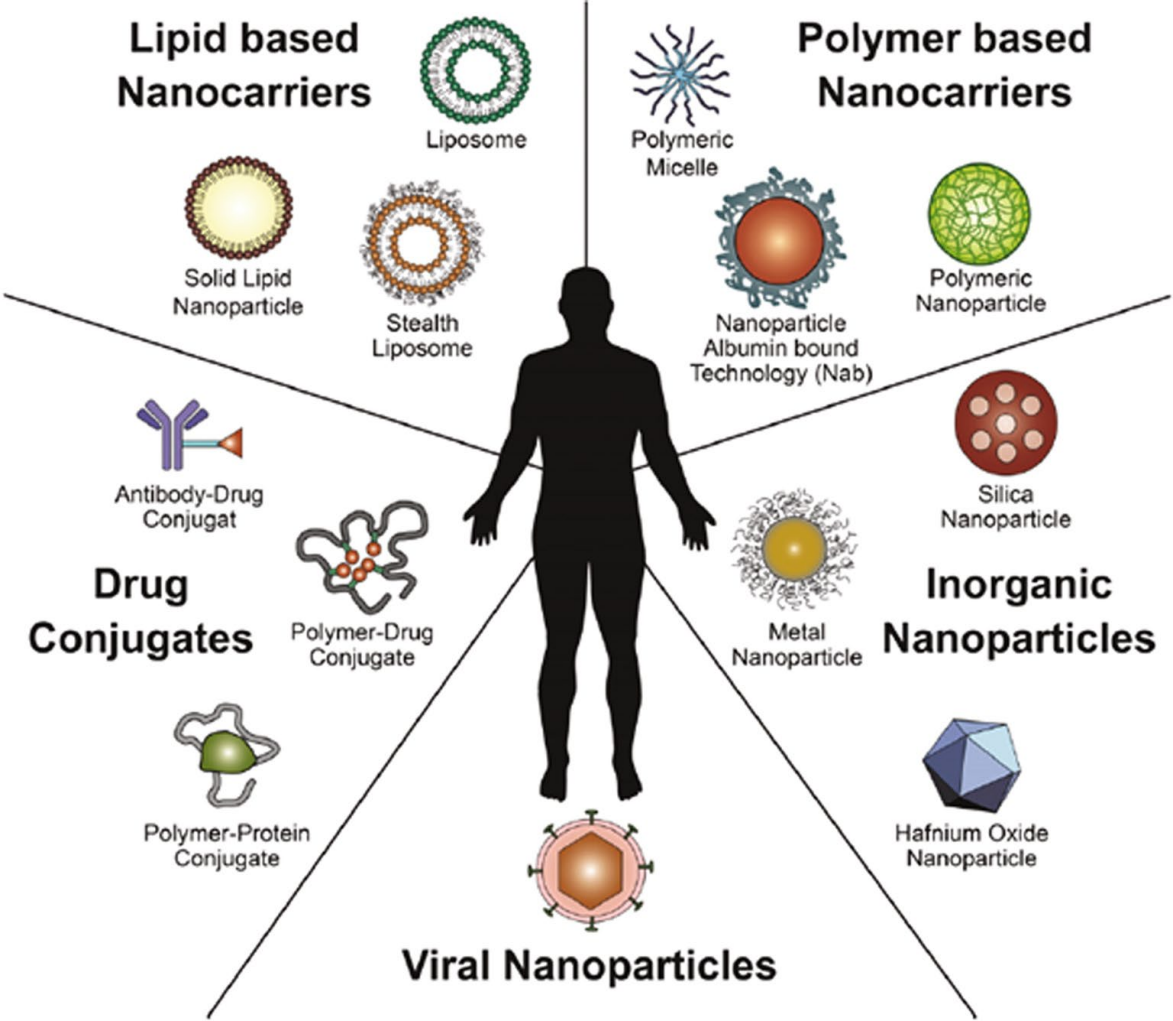
Overview of established nanomedicines in the clinic. This diagram shows an overview of the nanomedicines currently being investigated in the clinic for cancer treatment. Lipid-based, polymer-based, inorganic, viral, and drug-conjugated nanoparticles are examples of platforms that have been established in clinical research (Reproduced with permission from [3])

Since the characterization of cancer via these hallmarks of cancer, new methods for treatment have been investigated. Nanomedicine can be defined as nanotechnology, or the use of materials between 1 and 100 nm, applied to health and medicine [3]. Nanomedicine is an emerging method for treating cancer. Current problems in treating cancer include low specificity, rapid drug clearance and biodegradation, and limited targeting [4]. The properties of nanocarriers, including their nanoscale sizes, high surface-to-volume ratios, favorable drug release profiles, and targeting modifications, can allow them to better reach target tumor tissue and release drugs in a stable, controlled manner [3]. Nanocarriers can accumulate in leaky vasculature, which is a characteristic of tumor tissue, in an effect known as the enhanced permeability and retention effect (EPR) effect [5]. Use of internal and external stimuli as well as targeting modifications may assist in the controlled release of the drug to ensure specific toxicity to the tumor tissue, while sparing normal tissue. The poor solubility of small molecule drugs often restricts their delivery to the tumor, and therefore encapsulating the drugs in nanocarriers may facilitate travel through the bloodstream, thus preventing rapid clearance and improving bioavailability. The potential of nanomedicines can be further extended to early detection of cancers as well as combination therapies that can start treating tumors earlier and more effectively. Currently, a wide variety of platforms are being investigated as nanocarriers for cancer treatment, including lipid-based, polymer-based, inorganic, viral, and drug-conjugated nanoparticles (Fig. 1). Several of these same platforms have also been approved for use in the clinic (Table 1).

**Table 1:** Currently approved nanomedicines in the clinic

Year approved	Name	Type	Active drug	Diameter (references)	Type of cancer
Japan (1994)	Zinostatin stimalamer	Polymer protein conjugate	Styrene maleic anhydride neocarzinostatin (SMANCS)	*	Renal cancer
FDA (1995) EMA (1996)	Doxil/caelyx	Liposome (PEGylated)	Doxorubicin	80–90 nm [82]	HIV-associated Kaposi’s sarcoma, ovarian cancer, metastatic breast cancer, multiple myeloma
FDA (1996)	DaunoXome	Liposome (non-PEGylated)	Daunorubicin	45 nm [83]	HIV-associated Kaposi’s sarcoma
Taiwan (1998)	Lipo-Dox	Liposome	Doxorubicin	180 nm [84]	Kaposi’s sarcoma, breast and ovarian cancer
FDA (1999)	DepoCyt	Liposome	Cytosine arabinoside (cytarabine)	10–20 µm [84]	Neoplastic meningitis
EMA (2000)	Myocet	Liposome	Doxorubicin	190 nm [84]	Breast cancer
FDA (2005) EMA (2008)	Abraxane	Nanoparticle albumin bound	Paclitaxel	130 nm [27]	Advanced non-small-cell lung cancer, metastatic pancreatic cancer, metastatic breast cancer
FDA (2006)	Oncaspar	PEG protein conjugate	l-Asparaginase	50–200 nm [84]	Leukemia
South Korea (2007)	Genexol-PM	PEG-PLA polymeric micelle	Paclitaxel	20–50 nm [85]	Breast cancer, Lung cancer, Ovarian cancer [126]
EMA (2009)	MEPACT	Liposome (non-PEGylated)	Mifamurtide	*	Osteosarcoma
EMA (2010)	NanoTherm	Iron oxide nanoparticle	–	20 nm [86]	Thermal ablation glioblastoma
FDA (2012)	Marqibo	Liposome (non-PEGylated)	Vincristine	100 nm [87]	Philadelphia chromosome negative acute lymphoblastic leukemia
FDA (2015)	MM-398 (Onivyde)	Liposome (PEGylated)	Irinotecan	80–140 nm [88]	Metastatic pancreatic cancer (2nd line)

* Data could not be found

This review will discuss the current use of clinically approved nanomedicines, the investigation of nanomedicines in clinical trials, and the challenges that may hinder development of the nanomedicines for cancer treatment. Several properties of nanocarriers make them suitable for delivering chemotherapeutic drugs to the target tumor tissue. Small molecule drugs like most chemotherapies have very short circulation half lives inside the body and nanoparticles can be made long-circulating thereby improving the bioavailability of these drugs and thus improving efficacy without the need for higher doses [4–6]. Nanoparticles also offer the opportunity to control the release of the encapsulated payload such that a high percentage of the trapped drug is released after the particles have reached their target tissue. This property of controlled release from nanoparticles can improve efficacy of the drugs while reducing off-target toxic effects [4–6]. Several of these virtues of nanoparticle-based drug delivery are discussed next.

## Nanocarrier properties

### Physico-chemical properties

The nanomaterials available for cancer research can be modified in size, shape, and surface characteristics for customization to treat specific tumors. Size is important for travel through the bloodstream and subsequent delivery of the nanocarriers to tumor tissue. While smaller nanoparticles can accumulate more easily in the leaky blood vessels of tumors than those that are larger, they can also extravasate into normal tissue. On the other hand, larger nanoparticles cannot extravasate as easily and thus their distribution in the bloodstream is highly variable [6]. The optimization of nanoparticle size may help improve specific uptake into tumor tissue. The shape of the nanocarriers may impact fluid dynamics and thus influence uptake. Currently, the use of spherical nanocarriers appears to be more common than that of the nonspherical variety due to challenges in synthesis and testing [7].

The charge of nanocarriers may also affect their stability and distribution in the blood. Positively charged nanoparticles were previously shown to most effectively target tumor vessels, but a switch to a neutral charge after extravasation allowed quicker diffusion of the nanoparticles to the tumor tissue [8]. The surface of the nanocarriers can also be modified with ligands that may prolong blood circulation and promote specific types of endocytosis and cellular uptake into tumor tissue.

### Solubility, degradation, and clearance

Drugs with poor water solubility may be eliminated from the bloodstream before reaching tumor tissue. The use of hydrophilic nanoparticles to encapsulate these drugs may improve their solubility, in turn improving their bioavailability in vivo and thus allow more effective delivery [3]. Coating nanoparticles with polyethylene glycol (PEG), a hydrophilic and non-ionic polymer, was shown to increase solubility and stability of nanoparticles [6]. Since PEG is uncharged, it does not disrupt the function of charged molecules, such as DNA [9].

The reticulo-endothelial system (RES) recognizes hydrophobic materials as foreign and eliminates them from the bloodstream, taking them up in the liver or the spleen. Foreign materials coated with opsonin proteins are more easily recognized by monocytes and macrophages [10]. Opsonization of hydrophobic molecules can reduce their ability to reach the tumor tissue and trigger inflammation following the secretion of cytokines from the phagocytic cells [10, 11]. PEGylated nanoparticles mask their hydrophobicity and therefore can prolong their circulation in the blood to allow adequate time to reach tumor tissue [9]. This reduction in clearance not only increases the half-life of the nanoparticle but also improves its bioavailability [9, 10]. This improvement in bioavailability allows the drug to circulate in the blood for a longer period of time, preventing degradation before reaching the tissue of interest. Additional modifications to the nanoparticle surface, such as ligands to overexpressed receptors, may assist in specific uptake of the drugs in the tumor. Controlled release mechanisms may also prevent non-specific delivery of the toxic drug to normal tissue.

### Targeting

**Fig. 2:**
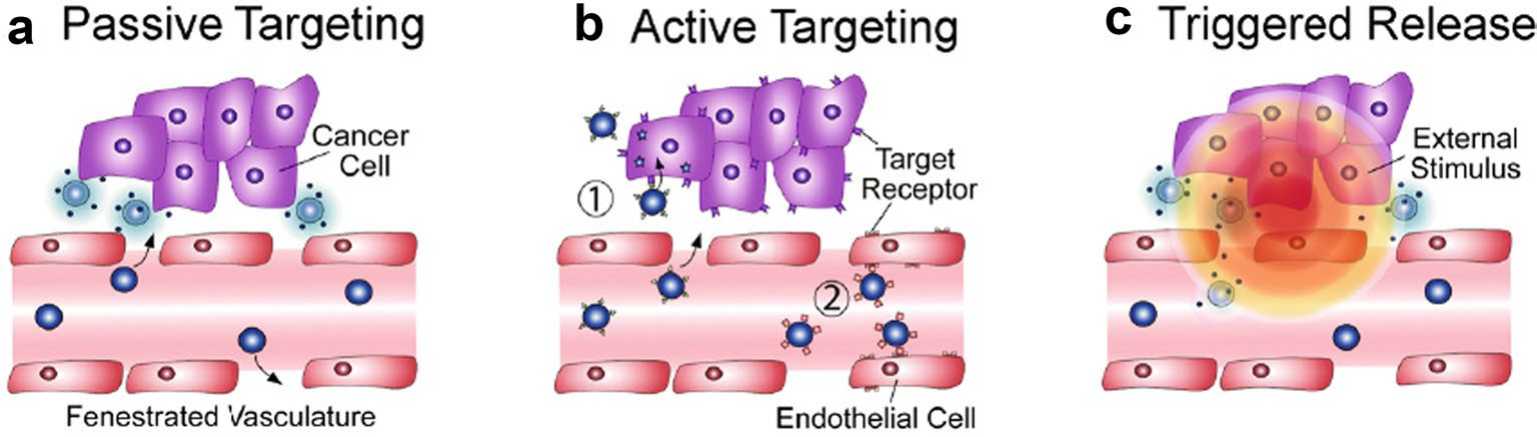
Types of targeting for nanoparticle delivery to tumor tissue. **a** Passive targeting relies on the leaky vasculature that is exhibited by tumors, allowing nanoparticles to travel through the fenestrations and reach tumors. **b** Active targeting can be used when nanoparticles have ligands on their surface that can recognize and bind receptors that are overexpressed on tumor cells. **c** Triggered release allows nanoparticles to congregate if exposed to an external stimulus such as a magnetic field or light (Reproduced with permission from [3])

Nanocarriers may be modified to utilize passive and active targeting mechanisms to reach tumor tissue (Fig. 2). The enhanced permeability and retention (EPR) effect allows nanoparticles to passively accumulate in the leaky blood vasculature exhibited by tumors without any surface modifications [3, 5, 6]. Passive targeting, however, cannot eliminate the potential of nanocarriers building up in tissues that normally have fenestrated blood vessels, such as the liver or the spleen [3]. Furthermore, the microenvironments of specific tumors vary and may pose as barriers for nanomedicine development.

Active targeting utilizes the attachment of ligands to surface of the nanocarriers [12]. These ligands have high specificity to receptors and other cancer-specific targets that are overexpressed on the surface of tumor cells, such as glycans [12]. Conjugation of these ligands may eliminate non-specific uptake of nanocarriers to tissue other than tumor tissue. Such ligands may include transferrin, folic acid, enzymes, engineered antibodies, and macromolecules like proteins and carbohydrates [3, 6]. The density of these ligands should be optimized to allow nanoparticles to avoid recognition by the RES and interaction with serum proteins, thus prolonging their blood circulation time [3].

### Stimuli-responsive and triggered release systems

The use of stimuli-responsive systems may reduce non-specific exposure to chemotherapeutic drugs (Fig. 2). Both internal and external stimuli can trigger the release of drugs by evoking a change in the nanocarriers. Changes in pH, redox, ionic strength, and stress in target tissues are examples of internal stimuli [3]. The differences in the pH of blood and intracellular organelles may allow nanocarriers to release drugs specifically when they reach tumor tissue [13]. pH responsive sodium alginate and hydroxyapatite bi-coated iron oxide nanoparticles were shown to exhibit a controlled drug release profile for the hydrophobic drugs curcumin and 6-gingerol and may offer a potential platform for cancer therapy [12]. Tumors typically have a hypoxic microenvironment with low oxygen and nutrient levels and thus high levels of reductive agents, such as glutathione [13–15]. Nanocarriers with disulfide bonds may be used to target these types of tissue. Nanocarriers with disulfide bonds can help carry out the redox reaction that oxidizes glutathione, which may increase cellular apoptosis [14].

External (physical) stimuli include temperature, light, ultrasound, magnetic force, and electric fields [3]. Hyperthermia, a temperature change with a range between 37 and 42 °C, may increase the permeability of blood vessels and enhance the delivery of nanocarriers, such as ThermoDox, which are responsive to these changes [16]. Use of near-infrared wavelengths may increase penetration into the body, compared to UV light, which is absorbed superficially by the skin, blood, and tissues less than 10 cm in depth [17]. Ultrasound systems may help diagnose cancers by triggering the release of contrast agents [18]. Magnetic and electric fields may allow nanoparticles to aggregate to specific sites [19].

The use of verteporfin (BPD) in nanoparticles is an example of nanoparticles that use a light triggered external stimulus. By itself, BPD is biocompatible. However, when exposed to infrared light, BPD produces reactive oxygen species (ROS) that can damage DNA, resulting in cell death. In a study by Konan-Kouakou et al., BPD loaded Poly(d,l-lactide-*co*-glycolide) (PLGA) nanoparticles were injected into mice with rhabdomyosarcoma (M1) tumors [20]. The mice were then exposed to laser light at 50 J/cm^2^ at 690 nm following a time delay (15, 30, and 60 min post injection) and evaluated for tumor growth for 20 days. After 20 days, the mice with shorter time delays (15 and 30 min) had more tumor-free mice (66 and 75% respectively) than those with the 60 min time delay (33%). These results were due to the rapid nanoparticle clearance in the blood stream. Another result of this rapid clearance was the reduction of side effects. The only side effect noted was photosensitivity of the skin. The 15 min mice had, at worst, visible pink erythema and a few broken blood vessels in some individuals, and these symptoms went away after 3 days. The 60 min mice had no side effects due to the rapid clearance on the nanoparticles.

### Combination therapy and theranostics

The ability of nanomedicines to carry multiple therapeutic agents may increase their ability to improve treatment. Co-loaded nanoparticles with bortezomib and doxorubicin were found to exhibit an antitumor synergistic effect on ovarian cancer [21]. Loading multiple siRNAs alone or together with other drugs may increase sensitivity of the tumor to the treatment [22, 23]. The use of stimuli-responsive systems with targeting ligands has also been investigated. An emerging method is the use of theranostics, which combines both the ability to diagnose and treat cancers. In theranostics, not only can drug release be monitored, but the effects of the drugs in the tumor tissue can also be visualized [24]. These abilities may open their potential to be used for personalized treatment [25].

**Fig. 3:**
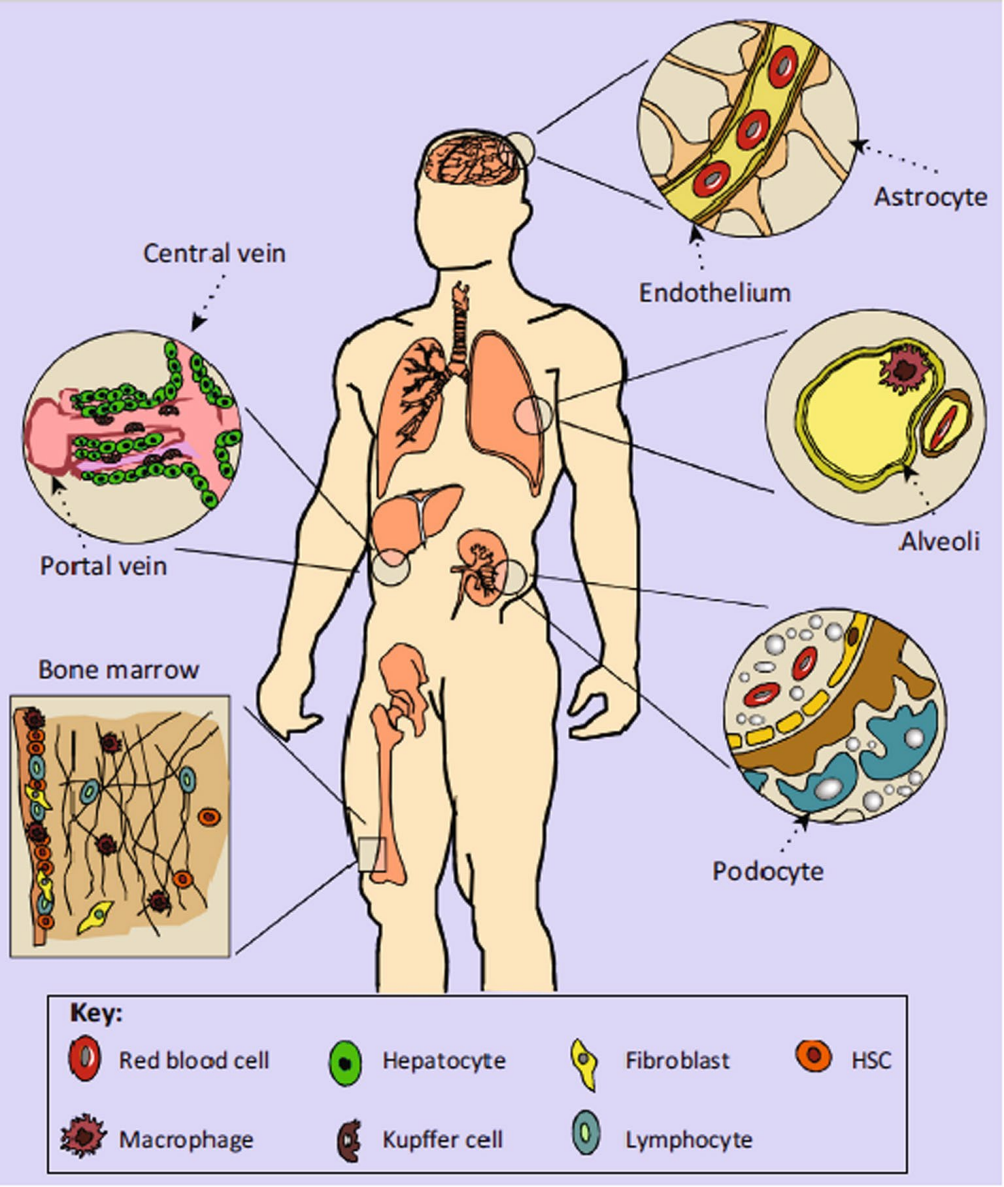
Organ systems that affect nanoparticle delivery. The method of entry affects circulation time, organ processing, and overall efficacy. Intravenously injected nanoparticles can extravasate from the bloodstream and enter organs such as the liver, spleen, bone marrow, and central nervous system. Nanomedicines that are administered orally can enter the gut and pass through the liver via the hepatic portal system. Inhaled nanomedicines may contact macrophages in pulmonary alveoli. Following circulation in organs, nanoparticles may encounter renal clearance in the kidneys (Reproduced with permission from [11])

Successful delivery of chemotherapeutic drugs is often dependent on the properties of the biological barriers involved (Fig. 3) in cancer. Next, we will discuss multiple biological barriers to effective drug delivery.

## Biological barriers to effective drug delivery

### Reticuloendothelial system

The reticuloendothelial system (RES), also known as the mononuclear phagocyte system (MPS), consists of both cellular and noncellular components. Phagocytic cells may bind nanoparticles and cause a release of cytokines, increasing nanoparticle clearance from the bloodstream and local inflammation of tissue [11]. Proteins, lipids, and other macromolecules may also bind to the surface of the nanoparticles and create a “biomolecular corona” around the nanoparticles. This effect may increase clearance from the bloodstream via recognition by the immune system or disrupt the ability of the nanoparticles to be internalized by the tissue of interest [26, 27]. Surface modifications of nanoparticles may permit escape from the RES and prolong their circulation time in the bloodstream, while preventing damage of normal tissue. Such modifications may involve zwitterionic ligands such as cysteine and glutathione or PEGylation [11, 26]. PEGylated nanoparticles can avoid recognition by RES, thus extending the time available for them to travel through the blood and reach target tumor tissue [9, 11].

Coating the nanoparticles with membranes derived from leukocytes or erythrocytes may enhance their self-recognition [11]. Use of ligands such as CD47-SIRPα to create signals may inhibit phagocytic clearance [28]. Spherical nanoparticles may congregate in the center of blood vessels and thus be less likely to extravasate via interactions with endothelial cells [29]. Use of disc-like nanoparticles may increase endothelial cell interactions and thus enhance their ability to extravasate into tumor tissues [30, 31]. Toxicity of the nanoparticles to organ systems of the RES should also be considered when designing their construct.

### Renal system

The kidney is responsible for filtering circulating blood, and therefore the barriers involved in kidney filtration need to be considered when designing nanoparticles. After passing through the fenestrated endothelium with 70–100 nm pores, nanoparticles must pass through the glomerular basement membrane, a thick layer of extracellular matrix that sits between the capillary endothelium and podocytes that permit clearance for 2–8 nm particles [11]. Openings of slit diaphragms that lie between epithelial podocyte extensions are regulated by proteins such as nephrin and CD2-associated protein, which generally allow the passage of water and small molecules [32].

Size, charge, and shape are all characteristics that affect the clearance of nanoparticles in kidneys. Spherical nanoparticles with diameters less than 6 nm were shown to have greater renal clearance than those with diameters greater than 8 nm [12]. The glomerular basement membrane is negatively charged, and therefore cationic nanoparticles of 6–8 nm exhibit greater clearance than those negatively charged or neutral of the same size [33]. Single-walled carbon nanotubes (100–500 nm length and 0.8–1.2 nm diameter) were found to pass through the fenestrated capillary endothelium by their long axis, despite their large molecular mass [34].

Although reducing nanoparticle size may enhance renal clearance, efficacy may be compromised. Multistage, biodegradable nanoparticles that dissolve into smaller particles that can be cleared by the kidney may be effective [35]. However, this effect may also pose a risk of nonspecific degradation, in which nanoparticles release drugs and other agents prior to arriving at target tumor tissue. Levels of the nano-encapsulated drugs must be sustained in the plasma for a chemotherapeutic effect, as well as eliminated from the bloodstream safely to limit potential long-term adverse effects. Personalized considerations must also be made for patients with renal deficiencies [11].

### Blood–brain barrier

The blood–brain barrier (BBB) poses a challenge for treating brain cancers, since it only permits passage of less than 2% of molecules, including ions, nutrients, specific peptides and proteins, and leukocytes [36]. The barrier consists of endothelial cells joined by tight junctions and enclosed by astrocytic cells, basal lamina, pericytes, and microglia. Current methods for increasing penetration may involve direct introductions into the brain such as intraventricular or intracerebral injection, infusion, and implantation and may increase toxicity risks and non-uniform drug dispersals [11].

Receptor-mediated endocytosis may be responsible for nanoparticle entry through the blood brain barrier [37]. This method relies on the conjugation of ligands or peptides to receptors that bind nanoparticles to the surface of the endothelial cells [11]. Surfactants such as poloxamer 188 or polysorbate 80 may be used to coat nanoparticles to increase adsorption of serum proteins such as apolipoproteins E or A–I and thus enhance penetration through the BBB [38]. Covalent attachment of apolipoproteins to human serum albumin-coated nanoparticles may also improve their ability to cross the BBB [39]. Use of targeting ligands that bind to receptors on endothelial cells, such as transferrin, lactoferrin, and low-density lipoprotein receptors, may also promote BBB penetration [37, 40, 41].

Nanoparticle size and charge are essential considerations for passing through the blood brain barrier. One study demonstrated that nanoparticles with diameters of 20–70 nm are preferential for transport [42]. Neutral and anionic nanoparticles were found to result in less neurotoxicity than cationic nanoparticles for an in situ perfusion study of rat brains [43].

Although reaching the brain may be a challenge for nanotherapy targeted for brain cancers, nanomedicines can also unnecessarily accumulate in the brain and cause toxic short-term and long-term effects. Nanoparticles containing metals such as copper, silver, and aluminum may degrade the BBB and cause neurotoxicity [44]. Microglial cells coordinate inflammatory signals and responses in the brain, and they can be activated by nanoparticle proximity and uptake, resulting in increased levels of inflammation [45]. Designing therapies that reduce activation of microglial cells may be useful in reducing neurotoxic effects [11].

### Pathophysiological barriers in cancer

Tumor tissue is often characterized by leaky vasculature rich in fenestrations and poor in pericyte coverage. This phenomenon, known as the enhanced permeability and retention (EPR) effect, has been utilized for passive targeting of nanoparticles to tumor tissue, but deeper penetration into the tumor is frequently restricted due to heterogeneity of the tumor microenvironment [46]. Composition and structure of extracellular matrix, along with tumor vasculature, is highly variable and dependent on cancer type, location, and progression state, along with patient-specific characteristics [11].

Methods to increase nanoparticle penetration into the tumor bed are currently being investigated. Use of smaller nanoparticles may allow enhanced passage through the vasculature and deeper penetration into the tumor [47]. Multistage nanoparticles break down into their smaller components, and their use may extend circulatory half-life, while allowing enhanced penetration [35]. Nanoparticles with the ability to respond to environmental cues, termed “smart,” may also improve bioavailability and be an avenue for personalized treatment [48].

A wide variety of materials can be used to create nanoparticles for drug delivery. The most common materials include proteins, liposomes, polymers, polymer-lipid hybrids, dendrimers, hydrogels, phase change materials, and inorganic materials.

## Types of nanoparticles

### Protein-drug conjugated nanoparticles

Protein-drug conjugated nanoparticles consist of proteins directly conjugated to drug molecules. The link between the protein and the drug is typically biodegradable upon arrival in the cell. This can lead to premature release of the drug, as the biodegradable linker is readily destroyed by proteases and redox-altering agents found in blood. Recent platforms, such as protein-drug conjugated systems with linkers that stay in place until the nanoparticles reach the target site, have overcome this barrier. This system allows more precise and controllable delivery of the cytotoxic drug molecules, lessening the toxic effects of the treatment on the body [49]. Protein-drug conjugated nanoparticles are typically very small (10 nm), allowing the nanoparticle to have a long half-life in vivo and thus facilitating its delivery to the target tumor site [50]. More recently, antibody proteins have been added to protein-drug conjugated nanoparticles, improving their targeting ability. An inherent issue with protein-based nanoparticles is that the structural sensitivity of some drugs makes them difficult to attach to a protein base. Therefore, certain drugs may not be suitable for this nanoparticle delivery system. The linkers used in these systems may also be rapidly degraded by enzymes and agents commonly found in blood plasma, leading to premature activation of the drug and a decrease in circulation time, while increasing the drugs bioavailability [51].

### Liposomal nanoparticles

Liposome-based nanoparticles are spherical nanoparticles created via the use of lipid bilayers. These nanoparticles are created immediately when an amphiphilic lipid is added to water or other hydrophilic liquids, yielding spheres roughly between 50 and 500 nm. This procedure allows for the encapsulation of hydrophilic drug molecules by simply dissolving the drug in the liquid used for formation of the nanoparticles. Hydrophobic and amphiphilic drugs can be encapsulated by direct addition to the lipid solution before formation of the nanoparticles, leading to a layer of drug molecules between the lipid bilayer [49]. Common lab methods used to create liposomal nanoparticles include sonication, extrusion, reverse-phase evaporation, and solvent injection [52].

Thermosensitive liposomes may allow the release of encapsulated drugs at specific temperatures [53]. Use of these liposomes allows for targeted drug delivery to areas where energy sources, such as high-intensity ultrasound, microwaves, and radio frequencies, are applied. The use of high-frequency ultrasound, for example, has successfully been used in clinical applications of liposomes to enhance drug delivery [54].

Hydrophilic drug molecules do not easily diffuse through the lipid bilayer, while hydrophobic and amphiphilic drugs easily diffuse through the lipid bilayer, potentially leading to toxicity in normal tissues. Use of liposomes may alleviate the issue of hydrophilic drug delivery. Since the lipid bilayer is similar in structure to the cell membrane, the liposomal nanoparticle can either fuse with the cell membrane or lyse once combined with harsh environments inside the cell (i.e. inside peroxisomes and lysosomes), releasing the drug inside of the cell. Hydrophobic and amphiphilic drug molecules can be kept inside of the liposomal nanoparticle via the creation of a polymeric encasement around the lipid bilayer. Depending on the polymer used, the ability of the nanoparticle to easily fuse with the target cell can be hindered, and in such cases, an additional mechanism must be incorporated to release the drug payload. The use of polymeric coatings can also lead to other key benefits, such as increasing circulation time, improving bioavailability of encapsulated drug, increasing targeting efficiency, and altering surface charge of the liposomes [49].

### Polymeric nanoparticles

Polymeric nanoparticles are comprised of synthetic polymers, allowing customization of many key properties, such as molecular weight, biodegradability, and hydrophobicity. The synthesis of polymeric nanoparticles has also been well studied. A variety of methods have been designed to efficiently encapsulate drug molecules. Some examples of these methods include nanoprecipitation, electrospray, and emulsification. Polymeric nanoparticles are typically comprised of dense matrices with well-known degradation curves, making the drug release of these nanoparticles easier to manipulate in comparison to many other nanoparticle drug delivery systems [49].

Issues with using polymeric nanoparticles include limited shape and wide size distribution. Polymeric nanoparticles are typically spherical, while a wide variety of different sizes may be generated during synthesis. New techniques are currently being investigated to resolve these issues. The most recent approach is particle replication in nonwetting templates (PRINT). PRINT allows for the creation of uniform polymeric nanoparticles, allowing the customization of properties such as shape and size. This, the aesthetic properties of the nanoparticles, as well as the amount, rate, and pathway used for cellular uptake of the encapsulated drug molecule, may be tailored [55].

### Dendrimeric nanoparticles

Dendrimeric nanoparticles are comprised of dendrimers, which are spherical macromolecules with many branches originating from a central point. These nanoparticles are created layer by layer. The initial core of the dendrimer is incorporated onto the previous layer before branches are allowed to form. By using specific initiator cores, the size and degree of branching of the dendrimer can be easily manipulated, allowing for the polydispersity of the nanoparticle to be minimized. By carefully planning the scheme of cores and branching units, the molecular weight, size, branch density, flexibility, and water solubility can be specified [49].

### Hydrogels

Hydrogels are three-dimensional networks of cross-linked water soluble polymers that are able to retain fluid in large quantities. Most synthetic hydrogels are not biodegradable, but enzymatic, hydrolytic, and stimuli-responsive components can be added into the hydrogel matrix in order to create nanoparticles that are degradable under certain conditions. The uniqueness of hydrogels is in their fluid retainment—the high water content is very similar to biological tissues, reducing tension when introduced to tissue and making this nanoparticle biocompatible [56]. By controlling the amount of cross linking in the hydrogel matrix, the porosity of the hydrogel can be adjusted to control drug loading and release rates. Hydrogels also naturally have a positive surface charge and thus may strongly interact with the negatively charged cell membranes, increasing cellular uptake of drug payload. Since serum proteins also are negatively charged, however, hydrogels may aggregate to serum proteins, decreasing the circulation time of the nanoparticles [49].

### Other nanoparticle platforms

One well-characterized example of inorganic, metallic nanoparticles is gold. Gold has been widely used for both detection and direct cancer therapy with and without drug loading. The strong optical absorbance of gold allows it to be used for detection, while its photothermal properties make it suitable as an anticancer therapy. Complex structures with gold may be designed to increase the efficiency of the drug release. For example, drug molecules may be conjugated to the surfaces of the gold nanoparticles, while structures with hollow interiors may also be created to increase encapsulation efficiency. Many of these structures can be easily created and specifically designed, such as to include a wide range of optical properties. Controlled drug release is possible by adding a layer of thermoresponsive polymers on the nanoparticle surface. These polymers shrink in heat and expand in cooler temperatures, allowing for the control of the diffusion rate of the loaded drug particles. This technology can be combined with the photothermal properties of gold for novel drug delivery to specific regions. Shining a laser, for example, on the tumors to heat the gold nanoparticles when they are near the tumor site can increase effective drug loading while minimizing nonspecific toxicity [49].

Carbon nanotubes have also been analyzed for cancer treatment. These structures can bind to various biological materials and enter cells via endocytosis. Single walled carbon nanotubes (SWCNTs) form highly stable suspensions in physiological buffers, making them suitable for use in biological environments. These SWCNTs also bind to attached molecules via cleavable disulfide bonds, allowing for drug materials to be removed from the SWCNTs in the presence of certain enzymes [57]. Carbon nanotubes have also shown promise in potential treatments with their interesting optical properties. Like gold, carbon nanotubes can be used to photochemically damage tumor cells via photothermal and photodynamic therapy [58]. Carbon nanotubes can also be used to image cancer growth via resonance-enhanced Raman signatures [49, 59].

A third type of nanoparticle with cancer treatment potential are silver nanoparticles. While the exact mechanism of action of silver nanoparticles in cancer remains unclear, silver reacts with the acidic environment that is often found in cancer cells to create reactive oxygen species. These reactive oxygen species can induce damage to cellular materials and apoptosis occurs. Silver has also been observed to inhibit vascular endothelial growth factor (VEGF), and thus possesses anti-angiogenic effects on cancer cells. In a study performed by Almajhdi et al., it was shown that the addition of silver nanoparticles to a nanofiber had an antitumor effect in vitro, with fibers containing 1% silver causing 8.8% of cancer cell death and an increase to 7% silver resulted in 67.6% cancer cell death. It was also seen that these nanofibers were able to create an inhibition zone of up to 10 mm (diameter) devoid of several bacterial strains [60], an advantage against infections if such a nanofiber was inserted into a tumor site. The main concern with the use of silver is toxicity, which can be overcome by creating nanoparticles with silver cores and a biocompatible shell. The outer layer of the nanoparticle is designed to break down in specific environments. Drug molecules can also be conjugated to this outer layer [61]. Evidence of the anricancer efficacy of silver nanoparticles in vivo using animal models of cancer is insufficient and warrants further studies.

Although there are multiple types of nanoparticles that can be created for cancer treatment, many of the treatments currently undergoing clinical trials are of the same nanoplatforms—mainly liposomal and to a lesser extent polymeric nanoparticles. This can be seen in Table 2, which shows several nanoparticle treatments that are in clinical trials in the United States. In order to further discuss clinical cancer nanomedicine, the approval of MM-398, a liposomal nanomedicine created to treat metastatic pancreatic cancer, is discussed. The drug’s preclinical and phase I, II, and III trials are reviewed in a case study format.

**Table 2:** Nanomedicines in clinical trials

Year	Name	Type of nano-particle	Active drug	Phase of trial	Size (nm)	Type of cancer	References
2017	NU-0129	Gold	Gold and siRNAs	I	*	Glioblastoma multiforme	[89, 90]
2017	MM-398	Liposome	Irinotecan	I, II, III	80–140	Small cell lung cancer, metastatic pancreatic adenocarcinoma, pediatric solid tumors	[70, 88, 91, 92]
2016*	Genexol-PM	Polymer	Paclitaxel	I/II	< 50	Gynecologic cancer, hepatocellular carcinoma, advanced breast cancer, non-small cell lung cancer	[93–96]
2016	CRLX101	Polymer	Camptothecin	I/II	30–40	Ovarian, rectal, renal, fallopian tube, lung (small cell and non-small cell) primary peritoneal, stomach, gastroesophageal, and esophageal cancers	[71, 72, 89, 97–101, 127]
2016	AuroLase	Silica-Gold	Gold	I	150	Prostate, head and neck, lung	[73, 102, 103]
2016	Vincristine sulfate liposome injection	Liposome	Vincristine	I/II	~ 100	Kaposiform hemangioendotheloma, Kasabach–Merritt Syndrome, tufted angioma, rhabdomyosarcoma, recurrent adult acute myeloid leukemia, lymphoblastic leukemia***, multiple lymphomas (non-hodgkin, follicular, mantle-cell, small-cell, and B-cell [Margional Zone]), Waldenstrom macroglobulinemia	[87, 104–108]
2016	Anti-EGFR immuno-liposome	Liposome	Doxorubicin	II	130–200	Breast cancer	[74, 109, 110]
2015	Mitoxantrone HCl liposome injection (plm60-s)	Liposome	Mitoxantrone	II	60	Breast cancer, diffuse large B-cell lymphoma, non-Hodgkin’s lymphoma, and peripheral T/NK lymphomas	[75, 111–114]
2014	CPX-351	Liposome		I	*	Acute myeloid leukemia	[115]
2014	LipoCURC	Liposome	Curcumin	I/II	*	Advanced cancer with failed standard of care therapy	[116]
2014	SGT-53	Liposome	Plasmid containing normal human wild type p53 DNA	I/II	*	Glioblastoma, neoplasm, metastatic pancreatic cancer	[117–120]
2013	LiPlaCis	Liposome	Cisplatin	I/II	*	Advanced or refractory solid tumours, metastatic breast cancer	[121, 122]
2012	PROMITIL	Liposome	Mitomycin-C	I	~ 90	Solid tumors	[123, 124]
2011	Abraxane	Protein-drug conjugate	Paclitaxel	II	~ 130	Breast cancer**	[125]

* Information not provided

** All patients are over the age of 65; breast cancers include estrogen receptor negative/positive, male breast cancer, Her2-negative/positive breast cancer, progesterone receptor positive/negative breast cancer, and several others

*** Patients were between age 1–21

## Case study: MM-398 clinical trials

**Fig. 4:**
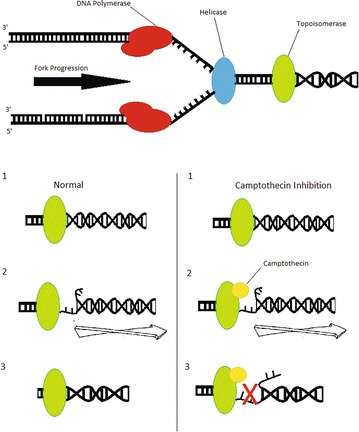
A schematic illustrating replication fork arrest by a drug-aborted topoisomerase I-DNA cleavable complex. In this model, the camptothecin trapped topoisomerase I-DNA cleavable complex is viewed as a bulky DNA lesion which arrests the replication fork by blocking the movement of replication machinery. This blockage also alters the physical state of the cleavable complex and possibly leads to fork breakage at the complex site. At low levels of cleavable complexes, when only one replication fork is arrested, continued replication by the other unimpeded fork on the same plasmid DNA leads to the formation of linearized replication products. The irreversible replication arrest and fork breakage may be the cause of camptothecin’s S-phase-specific cytotoxicity [62, 63]

**Fig. 5:**
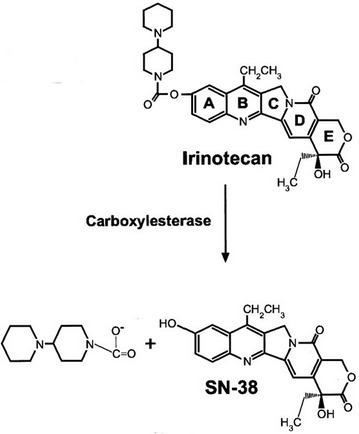
Metabolic pathway of irinotecan activation into SN-38 (Reproduced with permission from [81])

First approved in 1996, irinotecan (formerly known as CPT-11) is a semisynthetic analog of the cytotoxic alkaloid camptothecin. It is isolated from Camptotheca acuminata (family Nyssaceae), a tree indigenous to China. Camptothecin is known to have strong anti-tumor properties, and its analog irinotecan is typically used to combat colon and pancreatic cancers. Irinotecan is thought to have cytotoxic effects on cells in S phase, inserting itself into the DNA replication fork and effectively halting mammalian DNA topoisomerase I in its place, as seen in Fig. 4. This effect blocks DNA replication, inhibiting nucleic acid synthesis and inducing the DNA strands to break apart, ultimately causing cell death in proliferating cells [62, 63]. The active metabolite of irinotecan, SN-38, is in constant equilibrium with irinotecan (Fig. 5). Both substances have a pH-dependent equilibrium between their active lactone and inactive carboxylate forms. Although irinotecan and SN-38 both have the same DNA damaging ability, SN-38 is known to be roughly 100–1000 times more potent than irinotecan. Using unencapsulated irinotecan can thus lead to issues in toxicity and efficacy. Containing the irinotecan drug particles in liposomes may be a solution. Separation of the drug molecules from the inside of the body results in a lower plasma concentration (C_max_) and lower circulation time of the drug, both of these leading to a lower toxicity. A higher concentration of the drug is able to accumulate in the tumor tissues, possibly due to large and leaky vasculature in the tumor tissue [64]. Because of the pH equilibrium between the active and inactive forms of these drugs, a large amount of active SN-38 and irinotecan be delivered to the tumor site thanks to the acidic microenvironment of the tumor [65]. For these reasons, liposomal irinotecan (MM-398, formerly known as PEP02) has undergone preclinical and phase I–III testing and was recently approved for use as a second-line treatment for metastatic pancreatic cancer. These trials are outlined in the following sections.

### Preclinical studies

**Fig. 6:**
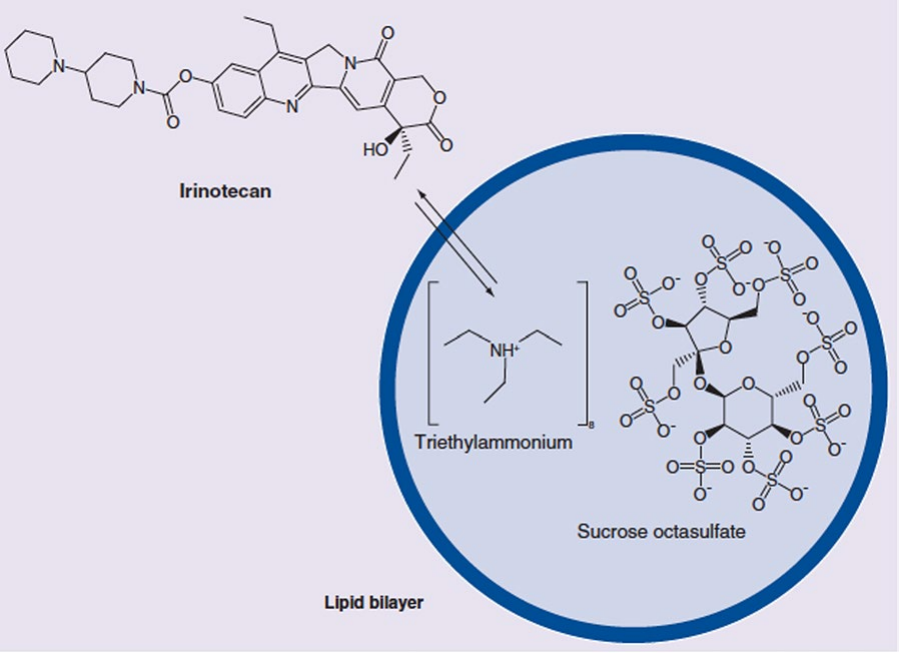
Depiction of the exchange of triethylamine for irinotecan, which forms a stable complex with sucrose octasulfate inside the liposome (Reproduced with permission from [66])

To improve the activation of irinotecan and address issues of overaccumulation in the blood, a liposomal encapsulation of irinotecan (MM-398) was designed to minimize the toxicity of the drug as well as achieve a higher efficacy [66]. Drummond et al. utilized highly charged amine groups on a multivalent anionic trapping agent (sucrose octasulfate [SOS] or linear poly(phosphate) [Pn]) to encapsulate irinotecan molecules in liposomes. The irinotecan molecules diffused into the liposomes readily; a triethylammonium (TEA) salt was used in a cation exchange mechanism to make up for the influx of drug molecules. Once in the liposome, the irinotecan molecules form a stable complex with the SOS or Pn matrix, effectively allowing for an extremely high drug-to-lipid ratio (109,000 molecules per particle) with a drug release half-life in the circulation of 56.8 h in Swiss Webster mouse models; these encapsulations will further be referred to as TEA-SOS and TEA-Pn. Synthesis of this stable irinotecan-sucrose octasulfate compound is depicted in Fig. 6. In mouse models, the maximum tolerated dose (MTD) was found to be much higher for nanoliposomal irinotecan (> 320 mg/kg) compared to free irinotecan (80 mg/kg). The efficacy of nanoliposomal irinotecan was also found to be greater than that of free irinotecan in cancer models. All mice with human breast (BT474) xenografts were completely cured of their tumors when treated with the encapsulated form, whereas “notable inhibition of growth” was observed in mice treated with the free form. Mice with human colon (HT29) xenografts treated with nanoliposomal irinotecan also showed significant improvement in survival compared to the free formulation. While the free irinotecan allowed the tumor volume to reach an average of roughly 2000 mm^3^ in about 35 days post-tumor implantation, all regimens involving nanoliposomal irinotecan resulted in an average tumor volume of under 100 mm^3^ in the same time span. After 66 days post tumor implantation, only one of the four nanoliposomal regimes led to a tumor volume similar to that of free irinotecan (25 mg/kg of liposomal irinotecan using a TEA-Pn encapsulation), resulting in an average tumor volume of 1750 mm^3^. The lowest tumor volume found after 66 days was 125 mm^3^, which was found in mice with a regimen of 50 mg/kg of liposomal irinotecan with a TEA-SOS encapsulation. None of the mice with colon cancer were found to be tumor free after undergoing the free irinotecan regimen, while 9.1% (n = 1) and 36.4% (n = 4) of mice undergoing the 25 and 50 mg/kg regimens respectively were tumor-free at the end of the study [67].

### Phase I

The goals of these studies were to find the dose-limiting toxicity (DLT), maximum tolerated dose (MTD) and pharmacokinetics (PK) of their respective treatments. The most common symptoms experienced by patients during the phase I trials include vomiting, diarrhea, neutropenia, and leukopenia. The former two symptoms were observed more commonly with low doses of liposomal irinotecan, and the latter two symptoms were more common with high doses of liposomal irinotecan [64, 68]. Summary tables of the most commonly observed symptoms from Chang et al. and Chiang et al. are seen in Table 3.

**Table 3:** Drug related adverse effects seen in phase 1 trials of liposomal irinotecan. Reproduced with permission from [64, 68]

	60 mg/m^2^ *N* = 1		120 mg/m^2^ *N* = 6		180 mg/m^2^ *N* = 4	
	All grade	G 3/4	All grade	G 3/4	All grade	G 3/4
	*N* (%)	*N* (%)	*N* (%)	*N* (%)	*N* (%)	*N* (%)
A						
Diarrhoea	1 (100)	0 (0)	6 (100)	2 (33.3)	4 (100)	1 (25)
Vomiting	1 (100)	0 (0)	5 (83.3)	4 (66.7)	2 (50)	2 (50)
Nausea	1 (100)	0 (0)	4 (66.7)	2 (33.3)	2 (50)	1 (25)
Alopecia	0 (0)	NA	3 (50)	NA	3 (75)	NA
Fatigue	1 (100)	0 (0)	3 (50)	1 (16.7)	1 (25)	0 (0)
Leukopenia	0 (0)	0 (0)	2 (33.3)	1 (16.7)	2 (50)	2 (50)
Neutropenia	0 (0)	0 (0)	2 (33.3)	1 (16.7)	2 (50)	2 (50)
Weight decreased	1 (100)	0 (0)	2 (33.3)	0 (0)	1 (25)	0 (0)
Dizziness	0 (0)	0 (0)	2 (33.3)	0 (0)	0 (0)	0 (0)
Anaemia	0 (0)	0 (0)	1 (16.7)	0 (0)	1 (25)	0 (0)
Anorexia	0 (0)	0 (0)	1 (16.7)	0 (0)	1 (25)	0 (0)
Electrolyte imbalance	0 (0)	0 (0)	1 (16.7)	0 (0)	1 (25)	0 (0)

AE	Total (*N* = 16)	60 mg/m^2^ *N* = 3	80 mg/m^2^ *N* = 6	100 mg/m^2^ *N* = 5	120 mg/m^2^ *N* = 2
	All grade	Grade 3–4			
B					
Anemia	7 (43.8%)	0	0	2 (40%)	0
Leukopenia	6 (37.5%)	0	0	2 (40%)	1 (50%)
Neutropenia	6 (37.5%)	1 (33.3%)	1 (16.7%)	2 (40%)	1 (50%)
Abdominal path	7 (43.8%)	0	0	1 (20%)	1 (50%)
Diarrhea	12 (75.0%)	0	1 (16.7%)	2 (40%)	2 (100%)
Nausea	13 (81.3%)	0	1 (16.7%)	0	0
Vomiting	12 (75.0%)	0	1 (16.7%)	0	0
Fatigue	8 (50.0%)	0	0	1 (20%)	0
Infection	6 (37.5%)	0	0	2 (40%)	1 (50%)
Anorexia	4 (25.0%)	0	0	1 (20%)	0
Hypoalbuminemia	4 (25.0%)	0	1 (16.7%)	0	0
Hypokalemia	8 (50.0%)	1 (33.3%)	2 (33.3%)	2 (40%)	1 (50%)
Hyponatremia	4 (25.0%)	0	0	1 (20%)	1 (50%)
Cough	5 (31.3%)	1 (33.3%)	0	0	0

A shows the adverse effects seen in Chang et al.’s study, while B shows the adverse effects seen in Chiang et al.’s study

*AE* adverse event

One study conducted by Chang et al. examined a cycle regimen of intravenous liposomal irinotecan in 90 min infusions every 3 weeks for a mean of 4 cycles (range 1–6). A total of 11 patients with solid refractory tumors participated in the study. Three different doses were given: 60 mg/m^2^ (one patient), 120 mg/m^2^ (six patients), and 180 mg/m^2^ (four patients). The MTD dose was found to be 120 mg/m^2^, and the most common treatment-related adverse events (AEs) at this dose were diarrhea (100% in all grades, 33% in grade 3/4) and vomiting (83.3% in all grades, 66.7% in grade 3/4); see Table 3A for a full list of observed symptoms. Only one patient was seen to have a reaction after infusion. This reaction involved chest tightness after the first 30 min of their infusion during cycle 2, but the patient’s vital signs were found to be stable. One treatment-related death was observed—a 67-year-old female patient with small cell carcinoma of the pancreas. This patient died of septic shock, disseminated intravascular coagulopathy and acute respiratory distress syndrome 7 days after first experiencing symptoms (8 days after her first dosing) [64].

Chiang et al. conducted a study to see the effects of giving infusions of liposomal irinotecan followed by infusions of 5-fluorouracil (5-FU) and leucovorin (LV) to 16 patients with solid refractory tumors. Cycles consisted of an infusion of intravenous liposomal irinotecan over a period of 90 min followed by 24 h infusions of 2000 mg/m^2^ 5-fluorouracil (5-FU) and 200 mg/m^2^ leucovorin (LV) on days 1 and 8 every 3 weeks. Four different concentrations of liposomal irinotecan were given: 60 mg/m^2^ (three patients), 80 mg/m^2^ (six patients), 100 mg/m^2^ (five patients), and 120 mg/m^2^ (two patients). The MTD was found to be 80 mg/m^2^, and the most common treatment-related AEs were nausea (81%), diarrhea (75%) and vomiting (69%) (see Table 3B for a full list of observed symptoms). There were no treatment-related deaths in this study [68].

### Phase II

The main goals of the phase II studies were to test percent dosages in humans and to test the efficacy, comparing liposomal irinotecan to the free form of the drug with an emphasis on toxicity.

**Fig. 7:**
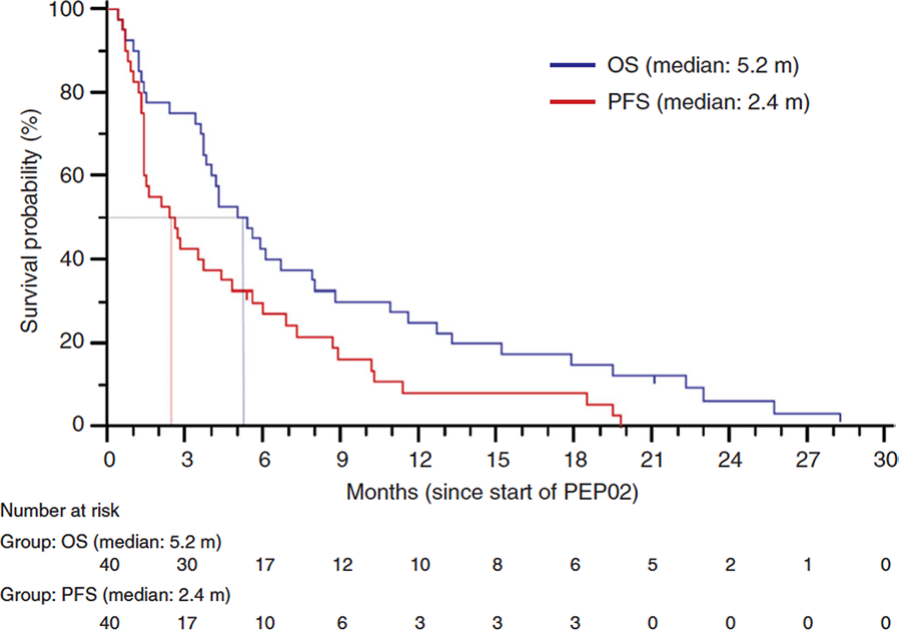
Kaplan–Meier curves of overall and progression-free survival. Abbreviations used are m for months, OS for overall survival, and PFS for progression-free survival (Reproduced with permission from [76])

One randomized study tested the objective response rate (ORR) of liposomal irinotecan in the second-line treatment of advanced oesophago-gastric (OG) cancer [69]. Patients with locally advanced or metastatic OG cancer were randomly assigned to one of three arms as a second-line treatment (having failed one prior chemotherapy). These arms were liposomal irinotecan 120 mg/m^2^, free irinotecan 300 mg/m^2^, and docetaxel (Taxotere) 75 mg/m^2^ every 3 weeks, with a total of 44 patients in each arm. Patients in each arm were equally represented in terms of age, sex, country of origin, previous treatment, and primary tumor site (gastric or GO junction). The study found that the time at which C_max_ (blood plasma concentration) was reached was much higher for liposomal irinotecan compared to normal irinotecan (10.2 compared to 2.1 h after infusion), showing that liposomal irinotecan stays active in the bloodstream for longer. The C_max_ value of formation of SN-38 was approximately 50% less after the infusion of liposomal irinotecan, showing that less of the drug was released prematurely when encapsulated. Although a smaller concentration of irinotecan was used in the nanoencapsulated arm than in the free irinotecan arm, the sum of the complete and partial responses was found to be twice as large in the nanoliposomal arm (13.6% compared to 6.8%), and the disease control rates were found to be comparable in both arms (59.1% in the nanoliposomal irinotecan arm and 61.3% in the free irinotecan arm). Unexpectedly, liposomal irinotecan was also seen to have much higher rates of grade 3 or 4 events of diarrhea and nausea, as seen in Table 4. Lower rates were observed in other studies [70–75], though, and it is to be noted that all other toxicity events had similar rates of occurrence with free irinotecan. It is also to be noted that although the overall survival rate of liposomal irinotecan was the lowest of the three arms, the progression free survival rate was roughly 9 months longer than that of regular irinotecan and nearly four and a half months longer than that of docetaxel (Fig. 7) [69].

**Table 4:** Adverse effects of liposomal irinotecan. Reproduced with permission from [69]

	Most common grade 3–4 adverse events					
	PEPO2		Irinotecan		Docetaxel	
	*N*	%	*n*	%	*n*	%
Anaemia	2	4.5	2	4.5	3	6.8
Neutropaenia	5	11.4	7	15.9	2	2.6
Thrombocytopenia	1	2.3	1	2.3	0	0
Febrile neutropenia	3	6.8	5	11.3	2	2.6
Diarrhoea	12	27.3	8	18.2	1	2.3
Nausea	5	11.4	2	4.6	0	0
Vomiting	2	4.6	6	13.6	3	6.8
Anorexia	3	6.8	3	6.8	0	0
Fatigue	2	4.6	1	2.3	1	2.3

Most common grade 3–5 adverse events observed in a phase II study of liposomal irinotecan (PEP02), comprised of a liposomal irinotecan, irinotecan, and docetaxel arm

**Fig. 8:**
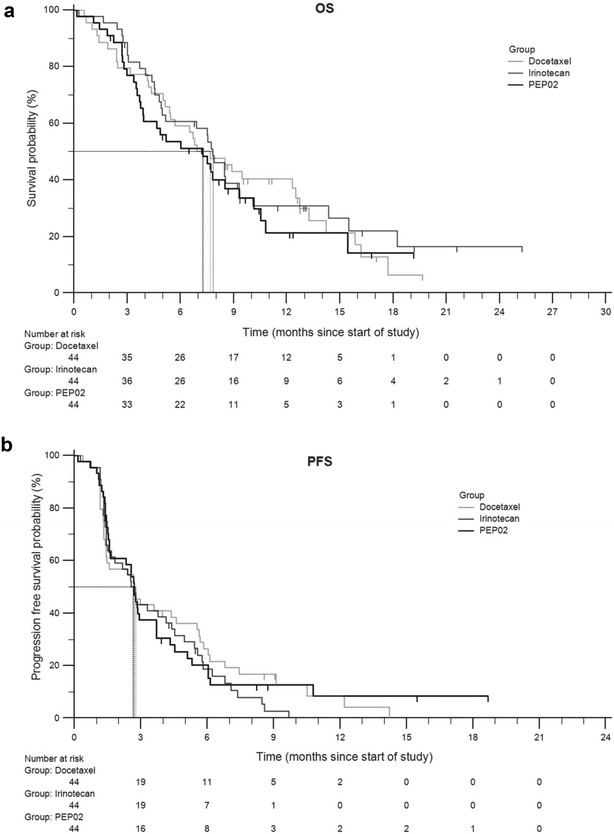
The Kaplan–Meier estimates of overall and progression-free survival (**a**, **b** respectively) in the intent-to-treat population for liposomal irinotecan (PEP02), irinotecan, and docetaxal (Reproduced with permission from [69])

A separate, multinational phase II study was conducted using liposomal irinotecan sucrosofate for patients with gemcitabine-refractory metastatic pancreatic cancer, the results of which led to a global phase III trial (NAPOLI-1) [76]. In this trial, a total of 40 patients were given 120 mg/m^2^ every 3 weeks; 27 of the 40 patients were able to stay at this dose, while 11 patients needed a reduced dose of 100 mg/m^2^ due to concerns of excess toxicity (mostly asthenia observed in US patients). The median progression-free and overall survival rates were 2.4 and 5.2 months respectively (Fig. 8). An objective response was seen in 7.5% of patients and disease control (partial response plus stable disease) was seen in 50% of patients. The most commonly seen adverse event was diarrhea (75% of patients), which the most commonly seen grade 3 or 4 events were neutropenia (30%) and leucopenia (25%). During this study three patients passed within the last 30 days of the last dose given during this study, all of which were due to neutropenia. As stated in Ko et al’s discussion, this highlights the need for watchful patient care, especially in such a fragile patient population, but with 75% of patients surviving at least 3 months, including 25% reaching the 1-year mark, liposomal irinotecan was approved to start phase III trials [76].

### Phase III

**Fig. 9:**
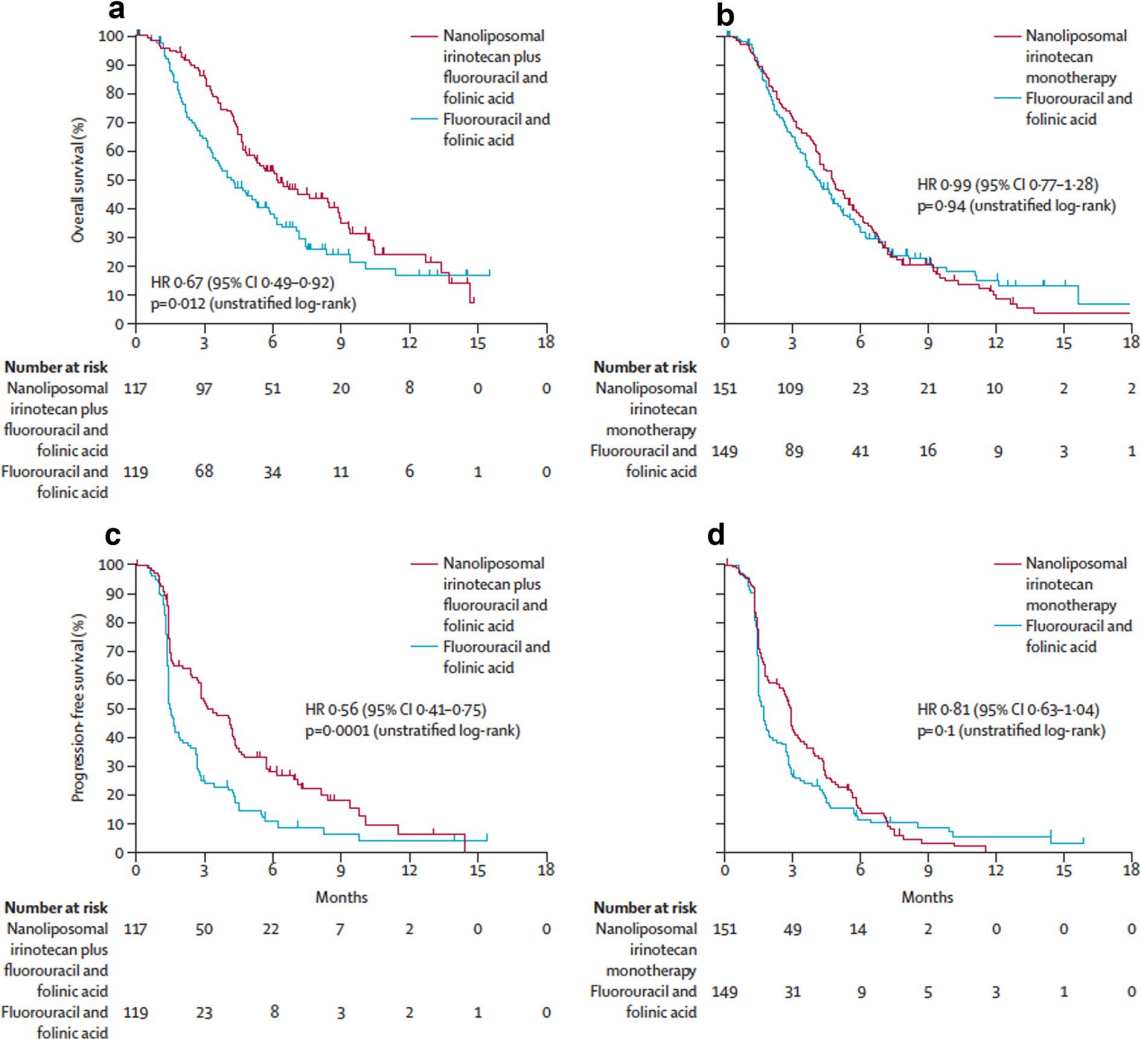
Kaplan–Meier survival analyses for phase III trial. *HR* hazard ratio. **a** Overall survival with nanoliposomal irinotecan plus fluorouracil and folinic acid versus fluorouracil and folinic acid. **b** Overall survival with nanoliposomal irinotecan monotherapy versus fluorouracil and folinic acid. **c** Progression-free survival with nanoliposomal irinotecan plus fluorouracil and folinic acid versus fluorouracil and folinic acid. **d** Progression-free survival with nanoliposomal irinotecan monotherapy versus fluorouracil and folinic acid (Reproduced with permission from [77])

In a global randomized phase III trial (NAPOLI-1), patients with metastatic pancreatic ductal adenocarcinoma were randomly assigned one of two arms as a second-line treatment following the first-line gemcitabine-based treatment [76, 77]. These two arms included a liposomal irinotecan monotherapy (120 mg/m^2^ every 3 weeks) and a control arm of fluorouracil (5-FU) given weekly as a 24-h infusion at 2000 mg/m^2^ and folinic acid (LV) at 200 mg/m^2^. An additional arm combining MM-398 (80 mg/m^2^) and 5-FU (2400 mg/m^2^) plus LV (400 mg/m^2^) as a biweekly treatment was added later. Patients in the three arms of the trial were evenly represented in age, sex, region, and Karnofsky performance status score. Patients who received the combination treatment (MM-398+5-FU/LV) had a median overall survival of 6.1 months (HR: 0.67; p = 0.012), in comparison to those given only MM-398 (4.9 months) and those given only 5-FU/LV (4.2 months) (Fig. 9). Thus, no statistically significant difference was observed between the monotherapy and the 5-FU/LV control for median overall survival. Improvements in median progression-free survival (3.1 vs. 1.5 months; HR: 0.56, p = 0.00001), objective response rate (16% vs. 1%), time to treatment failure (2.3 months vs. 1.4 months), and ≥ 50% decreases in CA 19–9 marker response (29% vs. 9%) were observed for patients who received the combination arm. Notable grade 3–4 adverse responses for the combination therapy were diarrhea (13%) and neutropenia (25%) (Table 5). Other symptoms including grade 3–4 responses are listed and compared for the three different treatments in Table 5.

**Table 5:** Adverse effects of MM-398. Reproduced with permission from [77]

	Nanoliposomal irinotecan plus fluorouracil and folinic acid combination therapy (n = 117)		Nanoliposomal irinotecan monotherapy (n = 147)		Fluorouracil and folinic acid control (n = 134)	
	Any grade	Grades 3–4	Any grade	Grades 3–4	Any grade	Grades 3–4
Diarrhoea	69 (59%)	15 (13%)	103 (70%)	31 (21%)	35 (26%)	6 (4%)
Vomiting	61 (52%)	13 (11%)	80 (54%)	20 (14%)	35 (26%)	4 (3%)
Nausea	60 (51%)	9 (8%)	89 (61%)	8 (5%)	46 (34%)	4 (3%)
Decreased appetite	52 (44%)	5 (4%)	72 (49%)	13 (19%)	43 (32%)	3 (2%)
Fatigue	47 (40%)	16 (14%)	54 (37%)	9 (6%)	37 (28%)	5 (4%)
Neutropenia*	46 (39%)	32 (27%)	37 (25%)	22 (15%)	7 (5%)	2 (1%)
Anaemia	44 (38%)	11 (9%)	48 (33%)	16 (11%)	31 (23%)	9 (7%)
Hypokalemia	14 (12%)	4 (3%)	32 (22%)	17 (12%)	12 (9%)	3 (2%)

Data are number of patients (%). The table shows grade 3 and 4 adverse events reported in ≥ 5% of patients whose treatment included nanoliposomal irinotecan with ≥ 2% incidence versus fluorouracil and folinic acid

* Includes agranulocytosis, febrile neutropenia, granulocytopenia, neutropenia, neutropenic sepsis, decreased neutrophil count, and pancytopenia

## Conclusions and future directions

The advent of nanomedicines represents significant advances in the field of drug delivery. The options for nanoparticle design and function are extremely varied and the list of potential applications continues to grow, to the point where the drug delivery system can be tailor-made to best suit the selected drug. However, it is important to remember that nanoparticle-based treatments are not miracle cures. They have both flaws and challenges to overcome. Selective targeting, while heralded as an improvement over non-encapsulated drugs, is a challenge unto itself. While many cancers overexpress surface proteins common in normal cells, overabundance of a specific surface protein is not enough to guarantee selectivity using targeted treatment. Ultimately, some of the drug will end up off-target, affecting non-cancerous cells. Choosing the right surface marker is critical for a targeted treatment to work. For liposomal irinotecan (MM-398), selectivity is achieved through the acidic tumor microenvironment. Irinotecan turns into its more active form, SN-38, in acidic environments. SN-38, then, disrupts the molecular machinery responsible for DNA replication. One could consider this a form of focused targeting: targeting only dividing cells in an acidic environment, such as those found in a tumor. However, tumor cells are not the only actively dividing cells in an acidic environment the body. Stomach epithelial cells are one such example. This may explain why most of the side-effects of MM-398 are digestive-related. Further, the tumor microenvironment is both heterogeneous and complex. The tumor is an amalgamation of both cancer and normal cells. Tumor cells invoke wound-repair pathways, recruiting basal laminal cells, blood vessel cells, and tumor-assisting macrophages (TAMs) to assist with growth and survival [2]. Due to cancer cells having a preference towards anaerobic metabolic pathways as well as the partial hypoxia of the tumor environment, pH gradients moving from extracellular to intracellular spaces tend to be reversed in tumor tissue when compared to normal tissue [78]. Differentiation between cancer cells within the same tumor can also occur. Due to genome instability, populations of different cancer cells can arise within a single tumor. As many as 20 driver mutations, and anywhere between 1000 and 100,000 point mutations can be found within individual cancers. Treatment may further increase the number of these mutations. “For example, gliomas that recur after treatment with the DNA alkylating agent temozolomide have been shown to carry huge numbers of mutations with a signature typical of such agents” [79].

There is also a reproducibility issue with nanoparticle production. Reproducible, large-scale synthesis of nanomedicines is still a challenge for the distribution of a homogeneous batch of nanomedicines, especially when considering that these nano-platforms often require specific conditions for production via self-assembly. Thorough characterization of these nanomedicines, at every stage of the production process must be enforced to ensure both reproducibility of synthesis and efficacy. Storage of these nanomedicines under appropriate conditions is also critical since colloidal instability can dramatically alter their performance in vivo. Ideal nanomedicines will have a modular design that can be easily scaled up for cGMP manufacturing and stored for a long time prior to use in patients.

Furthermore, the changes in legislation often occur at a rate different than the development of medicines in the laboratory. One organization, the Nanotechnology Characterization Laboratory, works with the FDA as bridge between scientists and regulatory committees to aid the review of nanomedicines [80] and has helped translation of some nanoplatforms.

Overcoming these challenges may seem like a herculean effort, but it is not impossible. There has been an overall shift in cancer research, from individual-based to a more collaborative approach that has helped achieve success. A complex problem requires a complex solution, and a multidisciplinary approach seems like the best option. Cross collaborations between theoretical and experimental scientists across academia, with the pharmaceutical industry, medical doctors and the regulatory agencies will help translate more findings from the lab to the clinic and usher in the next era of clinical cancer nanomedicines.

## Abbreviations

EPR: enhanced permeability and retention effect
PEG: polyethylene glycol
RES: reticulo-endothelial system
MPS: mononuclear phagocyte system
BPD: verteporfin
ROS: reactive oxygen species
PLGA: poly(d,l-lactide-*co*-glycolide)
BBB: blood–brain barrier
PRINT: particle replication in nonwetting templates
SWCNT: single walled carbon nanotube
VEGF: vascular endothelial growth factor
C_max_: plasma concentration
MM-398: liposomal irinotecan
SOS: sucrose octasulfate
Pn: linear poly(phosphate)
TEA: triethylammonium
MTD: maximum tolerated dose
DLT: dose-limiting toxicity
PK: pharmacokinetics
AE: adverse effects
5-FU: 5-fluorouracil
LV: leucovorin
ORR: objective response rate
OG: oesophago-gastric cancer
HR: hazard ratio
TAM: tumor-assisting macrophages
MDR: multi-drug resistance
